# Genome Sequence of Bacteriophage Infecting a Rare Pathogen, Pseudomonas luteola

**DOI:** 10.1128/mra.01113-21

**Published:** 2022-02-17

**Authors:** Nisha Rathor, Pooja Sachdeva, Rama Chaudhry

**Affiliations:** a Department of Microbiology, All India Institute of Medical Sciences, New Delhi, India; b Department of Microbiology, Chaudhary Charan Singh University, Meerut, Uttar Pradesh, India; Portland State University

## Abstract

This is a report of genome characterization of Pseudomonas phage AIIMS-Plu-RaNi infecting Pseudomonas luteola. The phage belonged to the family Siphoviridae with icosahedral head and tail with a genome of 46.6 kb, 64.45% GC with 68 open reading frames.

## ANNOUNCEMENT

The continuously increasing drug resistance of pathogenic microorganisms is creating the alarming situation to public health (1). Bacteriophages are attractive antibacterial agents other than antibiotics (2). Well-characterized phage genomes are the key to successful bacteriophage therapy (3). Here we report the genome characterization of Pseudomonas phage AIIMS-Plu-RaNi infecting the rare opportunistic pathogen Pseudomonas luteola. The P. luteola has been reported to cause many life-threatening infections including endocarditis, peritonitis, meningitis, septicemia, and brain abscesses etc. (4–9).

**FIG 1:**
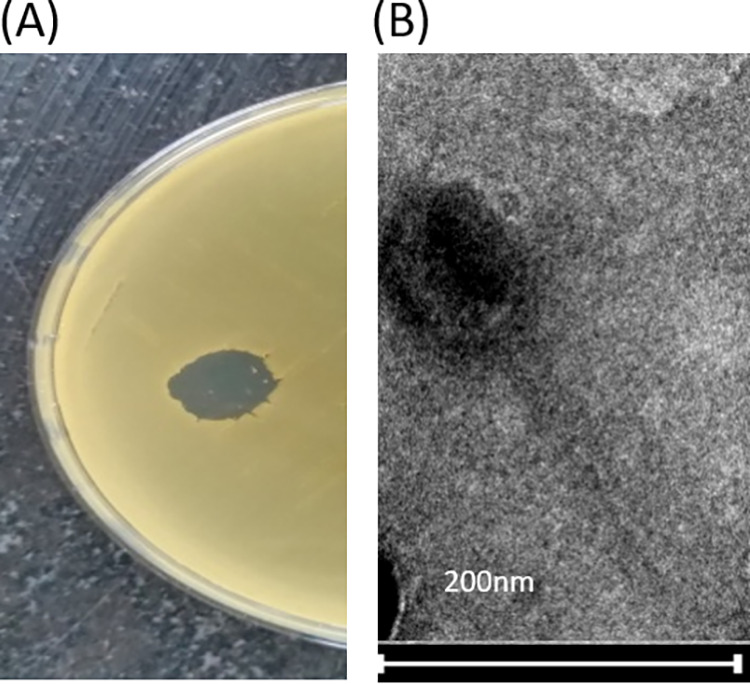
(A) The clear spot was produced after overnight incubation at 37°C, by the phage AIIMS-Plu-RaNi on the bacterial lawn of P. luteola, grown on MHA. (B) The icosahedral head and long tail of AIIMS-Plu-RaNi phage was imaged using the Transmission Electron Microscopy.

The P. luteola was isolated from the swabs, collected from the hospital floor ward at All India Institute of Medical Sciences (AIIMS), New Delhi, grown on Luria-Bertani agar, and identified by Matrix-Assisted Laser Desorption/Ionization Time of Flight spectrometry (10). The P. luteola-specific phage was isolated from the sewage water collected from the untreated sewage drainage of the residential area of AIIMS, New Delhi. In brief, the sewage water was treated with 1% chloroform, was incubated with log phase culture of P. luteola grown in Luria-Bertani broth (1:1, V/V), and incubated overnight at 37°C. The mixture was treated with 1% chloroform, centrifuged at 10,000 for 10 min and supernatant was collected. The supernatant produced a clear zone on spotting to P. luteola lawn on Mueller-Hinton Agar (MHA) plate after overnight incubation (11) (Fig. 1A), indicating presence of phage, named as AIIMS-Plu-RaNi. The phage particles were stained with 1% phosphotungstic acid (12) and showed the icosahedral head and tail on visualizing under TECNAI G20 HR-TEM Transmission Electron Microscope at Sophisticated Analytical Instrumental Facility, AIIMS, New Delhi (Fig. 1B).

For DNA isolation, the AIIMS-Plu-RaNi was concentrated with 1M NaCl and 10% Polyethylene glycol, and the DNA was extracted using phenol-chloroform -Isoamyl alcohol and precipitated by 95% ethanol (13).

The genome library was prepared using NEBNext® Ultra™ II FS DNA Library Prep Kit (Catalog: E7805S, New England Biolabs) as per the manufacture’s recommendation and sequenced by Clevergene Biocorp Pvt. Ltd., Bengaluru, India using Illumina HiSeq (2X150 bp run). All tools were run with default parameters unless otherwise specified. The 10,585,558 raw reads were generated and subjected to FastQC GPLv3 (14) and MultiQC GNU GPLv3 (15) for base call quality distribution, % bases above Q20, Q30, %GC, and sequencing adapter contamination. The low-quality bases and adapter sequences were removed by fastp v0.22.0 (16) and reads were assembled by Megahit GPLv3 (17).

The AIIMS-Plu-RaNi showed a genome of 46.6 kb with 64.45% GC content. The genome was analyzed by web BLASTn (18) for nucleotide similarity search and heuristic Hidden Markov Models using GeneMark v 4.28 (19) to predict the open reading frames (ORFs). The web BLASTp of the NCBI database was used for functional annotation of the ORFs (20).

The AIIMS-Plu-RaNi showed 95.18% nucleotide similarity with Pseudomonas phage PaMx11 with 91% coverage, and 68 ORFs were predicted.

### Data availability.

The phage AIIMS-Plu-RaNi sequencing raw reads are available through Sequence Read Archive of NCBI with BioProject number PRJNA772046 and run accession number SRR16530741. The annotated phage genome has been submitted to NCBI with accession number MZ926748.
